# Evaluating deliberative dialogues focussed on healthy public policy

**DOI:** 10.1186/1471-2458-14-1287

**Published:** 2014-12-17

**Authors:** John N Lavis, Jennifer A Boyko, Francois-Pierre Gauvin

**Affiliations:** McMaster Health Forum, Hamilton, ON Canada; Centre for Health Economics and Policy Analysis, McMaster University, Hamilton, Canada; Department of Clinical Epidemiology and Biostatistics, McMaster University, Hamilton, Canada; Department of Political Science, McMaster University, Hamilton, Canada; Department of Global Health and Population, Harvard School of Public Health, Boston, USA; School of Health Studies, Western University, London, Canada

**Keywords:** Deliberative dialogue, Knowledge translation, Evidence-informed decision-making, Public health policy

## Abstract

**Background:**

Deliberative dialogues have recently captured attention in the public health policy arena because they have the potential to address several key factors that influence the use of research evidence in policymaking. We conducted an evaluation of three deliberative dialogues convened in Canada by the National Collaborating Centre for Healthy Public Policy in order to learn more about deliberative dialogues focussed on healthy public policy.

**Methods:**

The evaluation included a formative assessment of participants’ views about and experiences with ten key design features of the dialogues, and a summative assessment of participants’ intention to use research evidence of the type that was discussed at the dialogue. We surveyed participants immediately after each dialogue was completed and again six months later. We analyzed the ratings using descriptive statistics and the written comments by conducting a thematic analysis.

**Results:**

A total of 31 individuals participated in the three deliberative dialogues that we evaluated. The response rate was 94% (N = 29; policymakers (n = 9), stakeholders (n = 18), researchers (n = 2)) for the initial survey and 56% (n = 14) for the follow-up. All 10 of the design features that we examined as part of the formative evaluation were rated favourably by all participant groups. The findings of the summative evaluation demonstrated a mean behavioural intention score of 5.8 on a scale from 1 (strongly disagree) to 7 (strongly agree).

**Conclusion:**

Our findings reinforce the promise of deliberative dialogues as a strategy for supporting evidence-informed public health policies. Additional work is needed to understand more about which design elements work in which situations and for different issues, and whether intention to use research evidence is a suitable substitute for measuring actual behaviour change.

## Background

Deliberative dialogues are a type of group process that can help to integrate and interpret scientific and contextual evidence for the purpose of informing policy development [1]. Such dialogues have recently captured attention because they have the potential to address several key factors that influence the use of research evidence in policymaking. These factors are: 1) interactions between researchers and policymakers; 2) timeliness of information; and 3) accordance between research evidence and the beliefs, values, interests or political goals and strategies of politicians, civil servants, and stakeholders [2]. Deliberative dialogues can support the prospects for research use by: 1) providing a forum for researchers and policymakers to interact; 2) identifying and interpreting the available research evidence about a high-priority policy issue on a timely basis; and 3) helping to identify accordance between research evidence and the beliefs, values, interests or political goals and strategies of politicians, civil servants and stakeholders.

While there is no singular approach to operationalizing a deliberative dialogue, some key features include: a) ensuring that the meeting environment is conducive to deliberation about a policy issue (e.g., adequate resources, a skilled facilitator, rules of engagement); b) a mix of participants that includes fair representation of all relevant interests; and, c) using research evidence to identify scientific uncertainties around issues and foster an equal knowledge base among participants [3, 4]. There is an emerging evidence base to support the "promise" of deliberative dialogues as a strategy for supporting evidence-informed public health policies. For example, while a systematic review about strategies to support evidence-informed decision-making about health systems did not identify any evaluations of the effectiveness of deliberative dialogues, the review did identify a variety of key design features, including consultation with all parties affected by the outcome, fair representation of researchers and stakeholders, high-quality syntheses of the scientific evidence, and skillful chairing [5]. Also, a formative evaluation of a policy dialogue involving policymakers, civil society groups and researchers from 20 low and middle-income countries found that the most highly valued design features included: pre-circulated evidence summaries; skilled facilitation; a Chatham House rule (prohibiting the attribution of particular comments in order to create a safe environment to deliberate about potentially contentious issues); and not emphasizing the need to achieve consensus since the dialogue brought together individuals with a range of accountabilities and decision-making authority [6]. Given the growing interest in using deliberative dialogues as a tool to increase the use of evidence in policymaking, it makes sense to strengthen the evidence base about them from a formative and summative perspective.

We conducted an evaluation of a series of three deliberative dialogues convened by the National Collaborating Centre for Healthy Public Policy (NCCHPP)^a^ in an effort to understand more about the key design features and impact of deliberative dialogues. The dialogues were part of a project that aimed to learn about overcoming the challenges in applying an evidence-informed approach to developing public health policies and to develop methodology to synthesize knowledge about policy measures in the field of public health. In general terms public policy refers to decisions, plans, or actions (i.e., interventions) that are undertaken to achieve a specific societal goal. Healthy public policies are the subset of public policies that do not have health as an explicit objective (e.g., healthcare policies) but that aim to achieve other objectives (e.g., social, economic or environmental objectives) while also enhancing population health and/or having a positive impact on the social, economic, and environmental determinants of health [7–10]. The dialogues in the current study focussed on policy measures to address obesity in Canada. The issue of obesity was chosen as a case study for understanding more about deliberative dialogues focussed on public health policies because it is a prominent issue in Canada and consensus does not exist as to the best policy measures to address the problem. The NCCHPP requested an independent evaluation of the three dialogues in order to inform future work aimed at supporting the use of research evidence in policymaking.

This paper summarizes our evaluation of the deliberative dialogue methodology applied by the NCCHPP. This paper is not intended to describe the outputs of the deliberative dialogues in terms of policy measures to address the featured policy issue (i.e., obesity in Canada). The specific objectives of our evaluation were to: 1) conduct a formative evaluation of the three deliberative dialogues, with a particular focus on participants’ views about and experiences with key design features of the dialogues; and, 2) conduct a summative evaluation of the three dialogues, with a particular focus on participants’ intention to use research evidence of the type that was discussed at the dialogues. A deliberative dialogue can contribute to making research available and meaningful to decision-makers and support action by stakeholders. A short-term measure of this may be "intention" to use research evidence from the deliberative dialogue [11]. According to the Theory of Planned Behaviour "intention" is an immediate precursor to "actual" behaviour [12]. The formative and summative findings reported in this paper are important contributions to understanding deliberative dialogues as a strategy to support evidence-informed policymaking about pressing health challenges.

## Methods

### Study participants

We surveyed all the individuals who participated in three deliberative dialogues convened by the NCCHPP. Each dialogue included 10–15 participants representing public policymakers (i.e., politicians, civil servants and/or political staff), stakeholders (i.e., public sector managers, professional association representatives, and consumer representatives) and researchers. Combined, the dialogue participants represent the key policymakers, stakeholders and researchers likely to be involved in or significantly affected by decisions related to addressing obesity. One dialogue was held in Vancouver, British Columbia and two held in Toronto, Ontario. The participants were purposively selected by the NCCHPP as part of the dialogue planning process to ensure each dialogue included appropriate representation from within the policy, practice and research communities in Canada. All participants were actively involved in effort to address obesity. Prior to the event the dialogue participants received background materials (including a summary of research evidence) about the featured policy issue and proposed policy measures. The deliberative dialogue itself was a one day event led by a trained facilitator and that followed the "Chatham House rule". The evidence brief and dialogue focussed on three policy measures (regulating televised advertising directed at children, regulating the labelling of nutritional information, and regulating the food available in schools). The study protocol was approved by the Hamilton Health Sciences/Faculty of Health Sciences Research Ethics Board (Project # 08–075).

### Questionnaire development and administration

We developed two questionnaires based on the research literature and pilot work. The first questionnaire was used to survey participants immediately after completion of the dialogue they participated in and the second questionnaire was used to follow-up on a small number of questions six months later. The relevant research literature included published descriptions of the attributes of deliberative dialogues that were identified as promising in a systematic review [5], and a manual describing how to produce survey questions based on the theory of planned behaviour [12, 13]. Pilot work included formative evaluations done of policy dialogues as part of the International Dialogue on Evidence-informed Action Project [14, 15]. A detailed description of how we developed the questionnaires has been published elsewhere [11].

The first questionnaire (available upon request from corresponding author) is divided into six sections with the first three sections comprising the formative evaluation, sections four and five the summative evaluation, and section six demographic questions about the participants’ role and background.

The first section includes questions that assess participants’ views about and experiences with key design features of the dialogues. Participants were asked to rate how useful they found ten specific design features on a scale from 1 (worthless) to 7 (useful), and to provide written comments. The second section includes an overall assessment of the deliberative dialogue. Participants were asked to rate how well the dialogue achieved its purpose and to provide written comments. The third section includes two questions that aim to gather comments about design features that should be retained or changed.

The fourth section includes rating questions based on the theory of planned behaviour, which suggests that behavioural intention is an immediate precursor of behaviour, and that behavioural intention is based on attitude toward the behaviour, subjective norms, and perceived behavioural control [12]. Based on this theory, we considered intention (along with the predictor variables) to use research evidence of the type that was discussed at the deliberative dialogues to be a measure of the dialogue’s impact. More specifically we measured: 1) "intention" using three items that together address the strength of the participants’ intention to use research evidence; 2) "attitude" using four statements that assess participants’ positive or negative evaluations of their use of research evidence; 3) "subjective norms" using four questions that assess participants’ evaluation of others’ opinions regarding their behaviour; and 4) "perceived behavioural control" using four items that assess participants’ evaluation of their own ability to use research evidence. The fifth section of the questionnaire includes two written response questions that aim to gather comments about future efforts to address the policy issue.

The second questionnaire (available upon request from corresponding author) that we developed includes two sections. The first section repeats the same request as the initial questionnaire for an overall assessment of the dialogue. The second section includes a written response question that aims to gather comments about efforts to address the featured policy issue, including what the participant personally had done.

Participants received the first questionnaire (along with a personalized cover letter, project summary, and a pre-stamped and addressed envelope) immediately following the dialogue they attended. Participants were asked to complete and return their completed questionnaire in a sealed envelope to a designated staff person. The designated staff person then sent the sealed and unopened envelopes to the principal investigator. If a participant was not able to complete their questionnaire immediately following the dialogue, then a staff person asked them to complete and mail the questionnaire as soon as possible. We mailed the second questionnaire (along with a personalized cover letter, project summary, and a pre-stamped and addressed envelope) by post to those who completed the first questionnaire. For both questionnaires two reminders were sent by post to non-responders. All completed questionnaires were kept confidential by storing them in a locked cabinet. The data were entered into Excel spreadsheets and stored on a security-protected computer.

### Analysis

Our analysis included two main steps. First, we calculated descriptive statistics for all questionnaire ratings. Second, we carried out a thematic analysis of all the written comments by initially reviewing all the comments for emerging themes, and then categorizing the comments according to the main themes, as well as the ten key design features being evaluated. Two members of our research team independently reviewed and then came to agreement on the main themes and categorization of all the written comments.

## Results

A total of 31 individuals participated in the three deliberative dialogues that we evaluated. The response rate for our initial survey was 94% (n = 29). The most frequently self-identified role category was stakeholder (n = 18), followed by policymaker (n = 9) and then researcher (n = 2). The response rate for our follow-up survey was 56% (n = 14).

### Overall assessment

The mean overall assessment score (in response to the question - How well did the forum achieve its purpose?) was higher on the initial survey than at follow-up (5.3 vs. 4.2 on a scale from 1 (failed) to 7 (achieved)). However, a paired samples t-test found no significant difference between these scores (p ≥ .05). The comments pertaining to overall assessment generally reflected that participants thought the dialogue was useful, but that there were opportunities for improvement. For example, one participated noted that: "*there was good discussion and knowledge transfer, however, I would not characterize it as a "full" discussion of relevant considerations; some research evidence seemed lacking*". Another participant said that the "*discussion was full, but due to missing stakeholders some gaps remain within the discussion*".

### Views about and experiences with key design features

Table 1 describes the participants’ ratings of their views about the ten key design features that we evaluated. In general, all the design features were rated favourably among all respondent groups. The mean scores among policymakers were consistently lower than among stakeholders and researchers with the exception of the design feature of "did not aim for consensus". The mean scores among researchers were consistently higher than among policymakers and stakeholders. However, researchers were also the smallest represented group (n = 2). The lowest rated design features among all participants were "focused on different ways in which a policy issue could be framed" and "ensured fair representation among policymakers". The highest rated design features among all participants were "engaged one or more skilled facilitators to assist with the deliberations" and "allowed for frank, off-the-record deliberations by following the Chatham House rule".

**Table 1:** **Ratings of key design features by role categories**

Design feature ^1^	Role categories M(SD)			
	All (n = 29) ^2^	Policymakers (n = 9) ^3^	Stakeholders (n = 18) ^4^	Researchers (n = 2) ^5^
Addressed a policy issue faced in your jurisdiction	5.2(1.2)	4.9(1.2)	5.2(1.2)	6.5(0.7)
Focused on different ways in which a policy issue could be framed	4.9(1.3)	4.5(1.2)	4.9(1.3)	6.5(0.7)
Focused on alternative ways of addressing a policy issue	5.2(1.1)	4.6(1.1)	5.4(1.0)	6.0(0.0)
Was informed by pre-circulated packaged evidence summaries	5.7(1.0)	5.3(1.1)	5.8(1.0)	6.5(0.7)
Was informed by discussion about the full range of factors that can inform choices among alternative ways of framing and addressing a policy issue	5.2(1.4)	4.7(2.0)	5.3(1.1)	6.5(0.7)
Brought together all parties who could be affected by the outcome	5.4(1.7)	4.0(1.8)	5.8(1.4)	6.5(0.7)
Ensured fair representation among policymakers, those stakeholders who could be affected by the outcome, and researchers	5.0(1.4)	4.0(1.6)	5.4(1.0)	6.0(1.4)
Engaged one or more skilled facilitators to assist with the deliberations	6.1(1.0)	5.4(1.0)	6.3(0.9)	7.0(0.0)
Allowed for frank, off-the-record deliberations by following the Chatham House rule	6.0(1.3)	5.9(1.6)	5.9(1.2)	7.0(0.0)
Did not aim for consensus	5.9(1.0)	6.0(1.2)	5.7(1.0)	7.0(0.0)

^1^Questions pertaining to design features were on a scale from 1 (worthless) to 7 (useful).

^2^The number of participants who responded to each question ranged from 23 to 29.

^3^The number of policymakers who responded to each question ranged from 6 to 9.

^4^The number of stakeholders who responded to each question ranged from 13 to 18.

^5^The number of researchers who responded to each question was 2.

Table 2 provides illustrative examples of the written comments about the perceived usefulness of the design features and opportunities for improvement. There did not appear to be any difference in the nature of the comments arising from the three dialogues. The written comments across the dialogues reflected two main themes: 1) usefulness of the design features; and, 2) opportunities for improvement. The majority of comments about usefulness were positive reflections about the design features’ usefulness (n = 88). The comments categorized as opportunities for improvement (n = 113) related to several sub-themes: stakeholder involvement (n = 33), dialogue processes (n = 24), scope (n = 19), quality and relevance of content (n = 19), facilitation (n = 14), and evidence summaries (n = 2).

**Table 2:** **Illustrative examples of written comments about perceived usefulness of the design features**

Theme	Illustrative examples of written comments
Usefulness of the design features	• "it was valuable to read the material prior to the meeting – [the pre-packaged evidence summaries] were appropriate length"
	• "[not aiming for consensus was] very useful as we could just listen to teach other without judging"
	• "[the Chatham House rule] made me feel more comfortable being open"
	• "the facilitator was knowledgeable and drew more detail out when necessary"
Opportunities for improvement	
• Stakeholder involvement (n = 33)	• "a broader range of parties – possibly private sector representatives [should have been included]"
	• "[there was not] enough diversity between parties’ perspectives on the policy issues"
• Dialogue processes (n = 24)	• "recap or summarize the points raised"
	• "a wrap up summary with take away learning’s [should be] included"
• Scope (n = 19)	• "[the] issues discussed could have been more diverse [there was] lots of overlap in discussion"
	• "obesity [is] such a complex issue [and] this took much time to discuss - maybe a simpler issue?"
• Quality and relevance of content (n = 19)	• "[the policy issue could have been framed with] more examples from across Canada and internationally"
	• "more comprehensive background/policy materials [could be] researched"
• Facilitation (n = 14)	• "the facilitator needed to redirect the group back to the questions"
	• "a better intro about what we had to do even after intro I still wasn’t completely sure"

### Views about future dialogues

Twelve participants noted that the most important design feature to retain for future dialogues is skilled facilitation (n = 12). As one participant noted, "*the facilitator as an outside agent seemed to ensure an unbiased outcome – this is an effective approach".* Eight participants (n = 8) felt that bringing together all parties who could be affected by the outcome was most important as it allowed for a "*variety of perspectives [and] open dialogue*". The next most important features to retain were: the pre-circulated packaged evidence summaries (n = 6); alternative ways of addressing a policy issue (n = 4); and, the Chatham House rule (n = 4). Comments about features to change related to improved facilitation (n = 3) and better stakeholder representation (n=2).

### Views about using research evidence more generally

Table 3 describes the mean ratings of the theory of planned behaviour constructs. In general, the mean scores of each construct were high. The mean of the three items that measured intention to use research evidence was 5.8 on a scale from 1 (strongly disagree) to 7 (strongly agree).

**Table 3:** **Means and standard deviations of theory of planned behaviour constructs**

Constructs	All	Policymaker	Stakeholder	Researcher
^1^Behavioural intentions	5.8(0.7)	5.8(1.1)	5.7(0.8)	5.8(0.3)
^2^Attitudes	5.6(1.6)	6.0(1.0)	5.8(2.3)	5.0(1.4)
^3^Subjective norms	5.2(1.2)	5.5(2.0)	5.7(1.3)	4.5(0.4)
^4^Perceived behavioural control	5.1(1.2)	5.4(1.4)	4.9(1.5)	4.9(0.9)

^1^Mean of three items each with ratings on a scale from 1 (strongly disagree) to 7 (strongly agree).

^2^Mean of four items with ratings scales of 1 (harmful/bad/unpleasant/worthless) to 7 (beneficial/good/pleasant/useful).

^3^Mean of four items with ratings scales of 1 (I should not/strongly disagree) to 7 (I should/strongly agree).

^4^Mean of four items with ratings scales of 1 (strongly disagree/hard) to 7 (strongly agree/easy).

### Views about future efforts to address the featured policy issue (initial survey)

Most comments about actions that policymakers, stakeholders or researchers can do better or differently to address the featured policy issue were about collaborating and communicating with multiple stakeholders to inform policy development (n = 10). One comment that was broadly representative of the others was to *"communicate more often so we are aware of what is going on".* Other comments about future efforts to address the featured policy issue related to: applying an evidence-informed approach that incorporates various types of scientific evidence and contextual knowledge (n = 6); using a more methodical approach to policy change (n = 4); and supporting the evaluation of existing policy interventions to determine their effectiveness (n = 3).

Most comments about actions that participants can themselves do better or differently to address the featured policy issue pertained to applying an evidence-informed approach that incorporates various types of scientific evidence and contextual knowledge (n = 11). For example, one participant stated that he/she will "*work on better business case/economic/cost-benefit/ budget impact analyses for any given policy option*". Other comments about future efforts to address the featured policy issue related to: collaborating and communicating with multiple stakeholders to inform policy development (n = 8); and, revisiting the questions addressed during the deliberative dialogue to explore policy issues and ways to address it (n = 3).

### Views about efforts to address the featured policy issue (follow-up survey)

Four (n = 4) written comments from the follow-up survey described actions that policymakers, stakeholders and/or researchers have done better or differently to address the featured policy issue. Thirteen (n = 13) written comments pertained to actions that participants themselves have done better or differently since the dialogue to address the featured policy issue. These actions included: collaborating and communicating with multiple stakeholders to inform policy development (n = 5); being better informed about the policy issue (n = 3); applying an evidence-informed approach that incorporates various types of scientific and contextual evidence (n = 2); and connecting with individuals from the dialogue about matters related to the features policy issue (n = 2). Eight (n = 8) comments described a lack of awareness of any action taken.

## Discussion

### Principal findings

Four key findings are apparent from the formative and summative evaluations we carried out. First, the deliberative dialogues were rated favourably overall by participants. The mean overall assessment scores and written comments reflect that participants thought the dialogues generally achieved their purpose and were useful. Second, the ten key design features that we evaluated were rated favourably by participants. These same design features could be used in future dialogues to support a full discussion of relevant considerations (including research evidence) about a high-priority issue such as addressing obesity with public health policies. Third, the opportunities for improving the key design features that we identified represent ways in which these features can be "tailored" for dialogues that address public health policy issues. For example, dialogues that aim to address a policy issue of relevance to jurisdictions across Canada could ensure that the participants include appropriate cross-jurisdictional representation of policymakers, stakeholders and researchers. Another example is that the pre-circulated evidence brief could include more data and evidence from observational studies, administrative database studies and community surveys in order to compare the issue across jurisdictions. Our fourth key finding is that immediately following the dialogues the participants intended to use the research evidence of the type that was discussed, and within six months of the dialogues many had actually done so. This suggests that the deliberative dialogues may have had some impact on the development of policy measures to effectively address obesity in Canada.

### Strengths and limitations

Two key strengths of our evaluation study were the separation of tasks (with three members of our research team involved in the design and execution of the study protocol, as well as the analysis and interpretation of study results, but not in organizing the dialogues) and our use of a questionnaire that was based on a validated theory and that has demonstrated good psychometric properties [11]. Despite these strengths, it is also important to consider certain limitations of our study. First, since this study focussed on one particular policy issue, it is limited in terms of the generalizability of the results. Generalizing the findings of our evaluation to other policy issues or contexts should be done cautiously until more research is done that compares findings that emerge across other dialogues that address public health policies. Second, we recognize that the current study only provides a signal (i.e., an individual’s self-reported intention to use research evidence) that a policy change is possible as a result of the deliberative dialogue [16]. There are a range of influencing factors and intended effects of deliberative dialogue that have not been considered in this study. For example, if the aim of the dialogue is knowledge translation then intended effects could be considered at the individual level in the short-term, at the community/organizational level in the medium-term, or at the system-level in the longer-term [3].

### Future research

Our study suggests specific areas of research that could help strengthen the evidence base for deliberative dialogues. First, more formative evaluations that examine the usefulness of specific design elements in addressing specific issues in specific contexts should be conducted in order to learn more about what works in which situations and for different issues. Our current study only provides insight about the usefulness of specific design features in two policy contexts and for one issue. Second, summative evaluation measures based on validated theory such as the theory of planned behaviour should be further tested. For example, an assessment of criterion validity should be carried out to determine whether intention to use research evidence is a suitable substitute for measuring actual behaviour change. Future research could also employ analytical methods such as regression to explore the relationship between intention to use research evidence, the three factors that are known to explain this intention (attitude, subjective norms, and behavioural control), and the key role categories (policymakers, stakeholders, and researchers) of those who participate in deliberative dialogues focussed on health public policy. Such analysis would support the generalization of findings to other policy issues or contexts. Research in the aforementioned areas will contribute further knowledge about the promise of deliberative dialogues.

## Conclusion

The findings from the current study will inform the on-going development of more formative evaluations of deliberative dialogue in different contexts and for different issues, as well as more summative evaluations to determine whether deliberative dialogues can in fact influence public policy.

## Endnote

^a^The NCCHPP is one of six centres financed by the Public Health Agency of Canada. The six centres form a network across Canada; each hosted by a different institution and focused on a different topic in public health. They provide national focal points for knowledge exchange in key areas of public health. The goal of the NCCHPP is to support the efforts of the Canadian public health community to promote healthy public policy through the development of more informed strategies. The focus of the NCCHPP is public policy with a potential impact on social, economic and environmental determinants of health. The NCCHPP focuses on three main content areas: healthy public policy, public policy processes, and methodologies for knowledge synthesis and exchange. More information about the NCCHPP is available from: http://www.ncchpp.ca.
